# 2,5-Dimethyl­phenyl benzoate

**DOI:** 10.1107/S1600536809010976

**Published:** 2009-03-28

**Authors:** B. Thimme Gowda, Miroslav Tokarčík, Jozef Kožíšek, P. A. Suchetan, Hartmut Fuess

**Affiliations:** a Department of Chemistry, Mangalore University, Mangalagangotri 574 199, Mangalore, India; b Faculty of Chemical and Food Technology, Slovak Technical University, Radlinského 9, SK-812 37 Bratislava, Slovak Republic; c Institute of Materials Science, Darmstadt University of Technology, Petersenstrasse 23, D-64287 Darmstadt, Germany

## Abstract

In the title compound, C_15_H_14_O_2_, the plane of the central –C(=O)–O– group is inclined at an angle of 3.7 (2)° with respect to the benzoate ring. The two benzene rings are almost perpendicular, making a dihedral angle of 87.4 (1)°. In the crystal, mol­ecules are packed into infinite chains through weak C—H⋯π inter­actions.

## Related literature

For the preparation of the compound, see: Nayak & Gowda (2009 ▶); For related structures, see: Gowda *et al.* (2008**a* ▶,b* ▶).
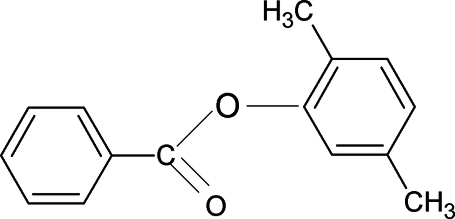


## Experimental

### 

#### Crystal data

C_15_H_14_O_2_
*M* *_r_* = 226.26
Monoclinic, 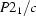

*a* = 8.1095 (4) Å
*b* = 9.8569 (4) Å
*c* = 15.8805 (10) Å
β = 105.617 (5)°
*V* = 1222.54 (11) Å^3^
*Z* = 4
Mo *K*α radiation
μ = 0.08 mm^−1^
*T* = 295 K
0.51 × 0.37 × 0.28 mm

#### Data collection

Oxford Diffraction Xcalibur diffractometer with Ruby (Gemini Mo) detector
Absorption correction: multi-scan (*CrysAlis RED*; Oxford Diffraction, 2009 ▶) *T*_min_ = 0.958, *T*_max_ = 0.982
29473 measured reflections
2330 independent reflections
1890 reflections with *I* > 2σ(*I*)
*R*_int_ = 0.025

#### Refinement

*R*[*F* ^2^ > 2σ(*F* ^2^)] = 0.037
*wR*(*F* ^2^) = 0.108
*S* = 1.07
2330 reflections
156 parameters
H-atom parameters constrained
Δρ_max_ = 0.14 e Å^−3^
Δρ_min_ = −0.10 e Å^−3^

### 

Data collection: *CrysAlis CCD* (Oxford Diffraction, 2009 ▶); cell refinement: *CrysAlis RED* (Oxford Diffraction, 2009 ▶); data reduction: *CrysAlis RED*; program(s) used to solve structure: *SHELXS97* (Sheldrick, 2008 ▶); program(s) used to refine structure: *SHELXL97* (Sheldrick, 2008 ▶); molecular graphics: *ORTEP-3* (Farrugia, 1997 ▶) and *DIAMOND* (Brandenburg, 2002 ▶); software used to prepare material for publication: *SHELXL97*, *PLATON* (Spek, 2009 ▶) and *WinGX* (Farrugia, 1999 ▶).

## Supplementary Material

Crystal structure: contains datablocks I, global. DOI: 10.1107/S1600536809010976/dn2434sup1.cif

Structure factors: contains datablocks I. DOI: 10.1107/S1600536809010976/dn2434Isup2.hkl

Additional supplementary materials:  crystallographic information; 3D view; checkCIF report

## Figures and Tables

**Table 1:** Hydrogen-bond geometry (Å, °)

*D*—H⋯*A*	*D*—H	H⋯*A*	*D*⋯*A*	*D*—H⋯*A*
C3—H3⋯CT1^i^	0.93	2.92	3.7477 (14)	148
C6—H6⋯CT1^ii^	0.93	2.91	3.6495 (14)	137

Symmetry codes: (i) 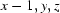; (ii) 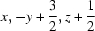. *CT*1 is the centroid of the benzoate ring C8–C13.

**Fig. 1.**
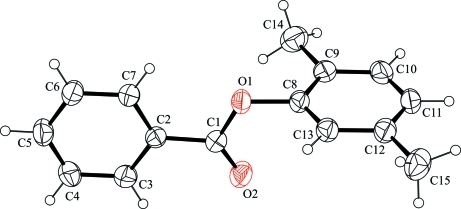
Molecular structure of (I) showing the atom labelling scheme. Displacement ellipsoids are drawn at the 30% probability level. H atoms are represented as small spheres of arbitrary radii.

**Fig. 2.**
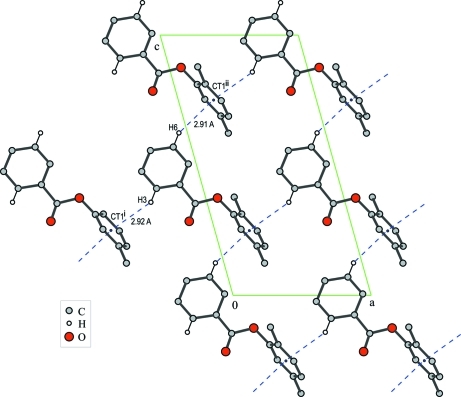
Part of the crystal structure of (I) viewed down the b axis. Infinite chains generated via C-H···π interactions are shown as dotted lines. CT1 is the benzoate ring (C8 to C13) centroid. H atoms not involved in hydrogen bonding were omitted. [Symmetry codes: (i) x-1,y,z; (ii) x,-y+3/2,z+1/2].

## supplementary crystallographic information

### Comment

As part of a study of the substituent effects on the solid state geometries of aryl benzoates (Gowda *et al.*, 2008*a, b*), in the present work, the structure of 2,5-dimethylphenyl benzoate (25DMPBA) has been determined. The structure of 25DMPBA (Fig. 1) is similar to those of 2,3-dimethylphenyl benzoate (23DMPBA) (Gowda *et al.*, 2008*a*), 2,4-dimethylphenyl benzoate (24DMPBA) (Gowda *et al.*, 2008*b*) and other aryl benzoates. The plane of central –O—C—O– group in 25DMPBA makes the dihedral angle of 3.7 (2)° with respect to the benzoate ring, compared with the value of 5.7 (1)° in the structure of 24DMPBA. The two aromatic rings in 25DMPBA form a dihedral angle of 87.4 (1)°, in comparison with the corresponding angle of 80.3 (1)° in 24DMPBA. The other bond parameters in 25DMPBA are similar to those in 23DMPBA, 24DMPBA and other aryl benzoates.

The molecules in the structure are packed into infinite chains through weak C-H···π interactions between some H atoms of the phenyl ring and the benzoate ring centroids. The first intermoleculer interaction includes the atoms C3, H3 and the benzoate ring centroid CT1 at the symmetry position (x-1,y,z). The second interaction includes C6, H6 and the benzoate ring centroid CT1 at the position (x,-y+3/2,z+1/2)(Fig. 2).

### Experimental

The title compound was prepared according to a literature method (Nayak & Gowda, 2009). The purity of the compound was checked by determining its melting point. It was characterized by recording its infrared and NMR spectra (Nayak & Gowda, 2009). Single crystals of the title compound were obtained by slow evaporation of its ethanol solution. The X-ray diffraction studies were made at room temperature.

### Refinement

H atoms were positioned geometrically and refined within a riding model with C—H distances of 0.93 or 0.96 Å and *U*_iso_(H) = 1.2 (1.5 for methyl) times *U*_eq_(C). Methyl groups were refined as freely rotating groups, using HFIX 137 command.

### Crystal data

**Table:**

C_15_H_14_O_2_	*F*(000) = 480
*M**_r_* = 226.26	*D*_x_ = 1.229 Mg m^−^^3^
Monoclinic, *P*2_1_/*c*	Mo *K*α radiation, λ = 0.71073 Å
Hall symbol: -P 2ybc	Cell parameters from 15569 reflections
*a* = 8.1095 (4) Å	θ = 3.2–29.5°
*b* = 9.8569 (4) Å	µ = 0.08 mm^−^^1^
*c* = 15.8805 (10) Å	*T* = 295 K
β = 105.617 (5)°	Block, colourless
*V* = 1222.54 (11) Å^3^	0.51 × 0.37 × 0.28 mm
*Z* = 4	

### Data collection

**Table:**

Oxford Diffraction Xcalibur diffractometer with Ruby (Gemini Mo) detector	2330 independent reflections
graphite	1890 reflections with *I* > 2σ(*I*)
Detector resolution: 10.434 pixels mm^-1^	*R*_int_ = 0.025
ω scans	θ_max_ = 25.9°, θ_min_ = 4.1°
Absorption correction: multi-scan (*CrysAlis RED*; Oxford Diffraction, 2009)	*h* = −9→9
*T*_min_ = 0.958, *T*_max_ = 0.982	*k* = −12→12
29473 measured reflections	*l* = −19→19

### Refinement

**Table:**

Refinement on *F*^2^	Primary atom site location: structure-invariant direct methods
Least-squares matrix: full	Secondary atom site location: difference Fourier map
*R*[*F*^2^ > 2σ(*F*^2^)] = 0.037	Hydrogen site location: inferred from neighbouring sites
*wR*(*F*^2^) = 0.108	H-atom parameters constrained
*S* = 1.07	*w* = 1/[σ^2^(*F*_o_^2^) + (0.051*P*)^2^ + 0.1667*P*] where *P* = (*F*_o_^2^ + 2*F*_c_^2^)/3
2330 reflections	(Δ/σ)_max_ < 0.001
156 parameters	Δρ_max_ = 0.14 e Å^−^^3^
0 restraints	Δρ_min_ = −0.10 e Å^−^^3^

### Special details

**Table:**

Geometry. All e.s.d.'s (except the e.s.d. in the dihedral angle between two l.s. planes) are estimated using the full covariance matrix. The cell e.s.d.'s are taken into account individually in the estimation of e.s.d.'s in distances, angles and torsion angles; correlations between e.s.d.'s in cell parameters are only used when they are defined by crystal symmetry. An approximate (isotropic) treatment of cell e.s.d.'s is used for estimating e.s.d.'s involving l.s. planes.
Refinement. Refinement of *F*^2^ against ALL reflections. The weighted *R*-factor *wR* and goodness of fit *S* are based on *F*^2^, conventional *R*-factors *R* are based on *F*, with *F* set to zero for negative *F*^2^. The threshold expression of *F*^2^ > σ(*F*^2^) is used only for calculating *R*-factors(gt) *etc*. and is not relevant to the choice of reflections for refinement. *R*-factors based on *F*^2^ are statistically about twice as large as those based on *F*, and *R*- factors based on ALL data will be even larger.

### Fractional atomic coordinates and isotropic or equivalent isotropic displacement parameters (Å^2^)

**Table:**

	*x*	*y*	*z*	*U*_iso_*/*U*_eq_	
O1	0.08573 (11)	0.73415 (10)	0.37466 (6)	0.0590 (3)	
O2	−0.16712 (13)	0.79648 (13)	0.28463 (7)	0.0766 (3)	
C1	−0.08701 (16)	0.75141 (14)	0.35356 (8)	0.0522 (3)	
C2	−0.16012 (16)	0.70922 (12)	0.42546 (8)	0.0478 (3)	
C3	−0.33645 (17)	0.71460 (15)	0.41159 (9)	0.0586 (4)	
H3	−0.4058	0.7447	0.3583	0.070*	
C4	−0.40821 (19)	0.67532 (17)	0.47672 (10)	0.0671 (4)	
H4	−0.5264	0.6778	0.4673	0.080*	
C5	−0.30493 (19)	0.63207 (16)	0.55630 (10)	0.0669 (4)	
H5	−0.3540	0.6058	0.6004	0.080*	
C6	−0.13073 (18)	0.62763 (15)	0.57060 (9)	0.0606 (4)	
H6	−0.0618	0.5993	0.6245	0.073*	
C7	−0.05770 (17)	0.66498 (13)	0.50552 (8)	0.0520 (3)	
H7	0.0605	0.6606	0.5151	0.062*	
C8	0.16784 (16)	0.77386 (14)	0.31055 (8)	0.0529 (3)	
C9	0.22655 (16)	0.90581 (15)	0.31172 (8)	0.0577 (3)	
C10	0.30805 (18)	0.93723 (17)	0.24763 (9)	0.0665 (4)	
H10	0.3487	1.0249	0.2449	0.080*	
C11	0.33025 (18)	0.84237 (19)	0.18820 (9)	0.0681 (4)	
H11	0.3845	0.8677	0.1460	0.082*	
C12	0.27415 (17)	0.71068 (17)	0.18959 (9)	0.0626 (4)	
C13	0.19194 (17)	0.67728 (15)	0.25320 (8)	0.0570 (3)	
H13	0.1533	0.5892	0.2567	0.068*	
C14	0.2076 (2)	1.00775 (18)	0.37817 (11)	0.0801 (5)	
H14A	0.0973	0.9975	0.3890	0.120*	
H14B	0.2175	1.0975	0.3566	0.120*	
H14C	0.2956	0.9938	0.4316	0.120*	
C15	0.2950 (2)	0.6074 (2)	0.12344 (11)	0.0871 (5)	
H15A	0.1904	0.6008	0.0776	0.131*	
H15B	0.3217	0.5207	0.1514	0.131*	
H15C	0.3862	0.6348	0.0992	0.131*	

### Atomic displacement parameters (Å^2^)

**Table:**

	*U*^11^	*U*^22^	*U*^33^	*U*^12^	*U*^13^	*U*^23^
O1	0.0521 (5)	0.0796 (6)	0.0484 (5)	0.0000 (4)	0.0188 (4)	0.0125 (4)
O2	0.0609 (6)	0.1169 (9)	0.0518 (6)	0.0068 (6)	0.0147 (5)	0.0194 (6)
C1	0.0531 (7)	0.0587 (7)	0.0451 (7)	−0.0021 (6)	0.0138 (6)	−0.0006 (6)
C2	0.0517 (7)	0.0480 (7)	0.0454 (6)	−0.0037 (5)	0.0159 (5)	−0.0056 (5)
C3	0.0530 (7)	0.0680 (9)	0.0545 (8)	−0.0003 (6)	0.0137 (6)	−0.0014 (6)
C4	0.0521 (8)	0.0831 (10)	0.0720 (9)	−0.0040 (7)	0.0269 (7)	−0.0051 (8)
C5	0.0696 (9)	0.0770 (9)	0.0643 (9)	−0.0056 (7)	0.0357 (7)	0.0002 (7)
C6	0.0664 (9)	0.0697 (9)	0.0487 (7)	0.0003 (7)	0.0204 (6)	0.0034 (6)
C7	0.0517 (7)	0.0576 (7)	0.0484 (7)	−0.0025 (6)	0.0163 (5)	−0.0028 (6)
C8	0.0455 (7)	0.0698 (8)	0.0432 (6)	−0.0006 (6)	0.0116 (5)	0.0114 (6)
C9	0.0513 (7)	0.0689 (8)	0.0492 (7)	−0.0028 (6)	0.0070 (6)	0.0075 (6)
C10	0.0558 (8)	0.0794 (10)	0.0607 (8)	−0.0133 (7)	0.0096 (7)	0.0171 (7)
C11	0.0494 (8)	0.1034 (12)	0.0545 (8)	−0.0048 (8)	0.0190 (6)	0.0147 (8)
C12	0.0476 (7)	0.0890 (11)	0.0518 (7)	0.0099 (7)	0.0144 (6)	0.0083 (7)
C13	0.0521 (7)	0.0665 (8)	0.0527 (7)	0.0014 (6)	0.0146 (6)	0.0075 (6)
C14	0.0914 (12)	0.0787 (11)	0.0666 (9)	−0.0073 (9)	0.0153 (8)	−0.0050 (8)
C15	0.0837 (12)	0.1139 (14)	0.0708 (10)	0.0228 (10)	0.0329 (9)	−0.0025 (10)

### Geometric parameters (Å, °)

**Table:**

O1—C1	1.3605 (16)	C8—C9	1.383 (2)
O1—C8	1.4136 (15)	C9—C10	1.3895 (19)
O2—C1	1.1977 (16)	C9—C14	1.495 (2)
C1—C2	1.4807 (17)	C10—C11	1.374 (2)
C2—C3	1.3880 (18)	C10—H10	0.9300
C2—C7	1.3885 (18)	C11—C12	1.378 (2)
C3—C4	1.372 (2)	C11—H11	0.9300
C3—H3	0.9300	C12—C13	1.3913 (19)
C4—C5	1.382 (2)	C12—C15	1.505 (2)
C4—H4	0.9300	C13—H13	0.9300
C5—C6	1.370 (2)	C14—H14A	0.9600
C5—H5	0.9300	C14—H14B	0.9600
C6—C7	1.3727 (18)	C14—H14C	0.9600
C6—H6	0.9300	C15—H15A	0.9600
C7—H7	0.9300	C15—H15B	0.9600
C8—C13	1.367 (2)	C15—H15C	0.9600
			
C1—O1—C8	116.19 (10)	C8—C9—C14	122.75 (13)
O2—C1—O1	122.74 (12)	C10—C9—C14	121.80 (14)
O2—C1—C2	125.28 (12)	C11—C10—C9	121.83 (14)
O1—C1—C2	111.98 (10)	C11—C10—H10	119.1
C3—C2—C7	119.61 (12)	C9—C10—H10	119.1
C3—C2—C1	118.43 (11)	C10—C11—C12	121.67 (13)
C7—C2—C1	121.96 (11)	C10—C11—H11	119.2
C4—C3—C2	119.83 (13)	C12—C11—H11	119.2
C4—C3—H3	120.1	C11—C12—C13	117.36 (14)
C2—C3—H3	120.1	C11—C12—C15	121.77 (14)
C3—C4—C5	120.05 (13)	C13—C12—C15	120.84 (15)
C3—C4—H4	120.0	C8—C13—C12	120.07 (14)
C5—C4—H4	120.0	C8—C13—H13	120.0
C6—C5—C4	120.36 (13)	C12—C13—H13	120.0
C6—C5—H5	119.8	C9—C14—H14A	109.5
C4—C5—H5	119.8	C9—C14—H14B	109.5
C5—C6—C7	120.09 (13)	H14A—C14—H14B	109.5
C5—C6—H6	120.0	C9—C14—H14C	109.5
C7—C6—H6	120.0	H14A—C14—H14C	109.5
C6—C7—C2	120.05 (12)	H14B—C14—H14C	109.5
C6—C7—H7	120.0	C12—C15—H15A	109.5
C2—C7—H7	120.0	C12—C15—H15B	109.5
C13—C8—C9	123.58 (12)	H15A—C15—H15B	109.5
C13—C8—O1	117.80 (12)	C12—C15—H15C	109.5
C9—C8—O1	118.54 (12)	H15A—C15—H15C	109.5
C8—C9—C10	115.44 (14)	H15B—C15—H15C	109.5

### Hydrogen-bond geometry (Å, °)

**Table:**

*D*—H···*A*	*D*—H	H···*A*	*D*···*A*	*D*—H···*A*
C3—H3···CT1^i^	0.93	2.92	3.7477 (14)	148
C6—H6···CT1^ii^	0.93	2.91	3.6495 (14)	137

Symmetry codes: (i) *x*−1, *y*, *z*; (ii) *x*, −*y*+3/2, *z*+1/2.
